# Plant Resistance Inducers against Pathogens in *Solanaceae* Species—From Molecular Mechanisms to Field Application

**DOI:** 10.3390/ijms17101673

**Published:** 2016-10-02

**Authors:** Erik Alexandersson, Tewodros Mulugeta, Åsa Lankinen, Erland Liljeroth, Erik Andreasson

**Affiliations:** 1 Department of Plant Protection Biology, Swedish University of Agricultural Sciences, P.O. Box 102, 23053 Alnarp, Sweden; erik.alexandersson@slu.se (E.A.); asa.lankinen@slu.se (Å.L.); erland.liljeroth@slu.se (E.L.); 2 Department of Zoological Science, Addis Ababa University, 1176 Addis Ababa, Ethiopia; mulugetatewodros@gmail.com

**Keywords:** β-aminobutyric acid (BABA), induced resistance, potato, *Solanaceae*, plant resistance inducers, PRI, phosphite, tobacco, tomato

## Abstract

This review provides a current summary of plant resistance inducers (PRIs) that have been successfully used in the *Solanaceae* plant family to protect against pathogens by activating the plant’s own defence. Solanaceous species include many important crops such as potato and tomato. We also present findings regarding the molecular processes after application of PRIs, even if the number of such studies still remains limited in this plant family. In general, there is a lack of patterns regarding the efficiency of induced resistance (IR) both between and within solanaceous species. In many cases, a hypersensitivity-like reaction needs to form in order for the PRI to be efficient. “-Omics” studies have already given insight in the complexity of responses, and can explain some of the differences seen in efficacy of PRIs between and within species as well as towards different pathogens. Finally, examples of field applications of PRIs for solanaceous crops are presented and discussed. We predict that PRIs will play a role in future plant protection strategies in *Solanaceae* crops if they are combined with other means of disease control in different spatial and temporal combinations.

## 1. Introduction and Background

Plant resistance inducers (PRIs) are agents that lead to improved protection to pathogen attacks by inducing the plant’s own defense mechanisms, so called induced resistance (IR). They are also referred to as plant resistance activators, plant defense activators and elicitors. PRIs are known to be effective against various pathogens, including viruses, bacteria, oomycetes and fungi attacking solanaceous plants. The IR phenomenon was described already in 1901 in Beauverie’s work “Essais d’immunisation des vegetaux contre les maladies cryptogamiques” [1], and was then followed by several studies in the early 1900s.

PRIs can be chemical compounds as well as microbial or plant extracts. However, they seldom lead to full pathogen control [2] and several factors influence the success such as plant genotype, developmental stage, environment, as well as timing and way of application of the PRI [3]. Importantly, all PRI strategies needs to be tested in an agricultural setting as many treatments have only been shown to be successful in more controlled conditions.

The effects of PRIs can be both local and systemic. In the latter case, increased resistance is seen in distal parts as a consequence of mobile signals in the plant leading to so called systemic acquired resistance (SAR; reviewed in [4]). Induced systemic resistance (ISR; reviewed in [5]), on the other hand, is triggered by the colonization of the roots by rhizobacteria or fungi. Whereas SAR is usually seen as salicylic acid (SA)-dependent, ISR is regarded as SA-independent and instead governed by jasmonic acid (JA) and ethylene (ET). Pioneer work exploring SAR was done in the solanaceou*s* species tobacco in the 1970s, when it was shown that injection of SA led to distal resistance to tobacco mosaic virus (TMV) [6].

**Figure 1:**
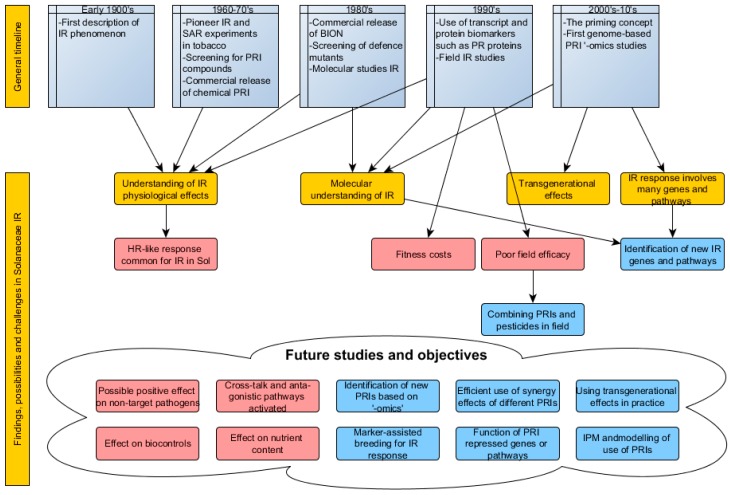
A time line of research methods and results as well as some future perspectives in the area of plant resistance inducers (PRI) and induced resistance (IR). SAR, systemic acquired resistance; PR, pathogenesis-related; HR, hypersensitive response.

The plant’s response to PRIs can also be associated with alterations in cell wall composition, production of phytoalexins and anti-microbial protein as well as to hypersensitive response (HR). The HR is in turn linked to the production of reactive oxygen species (ROS) and nitric oxide (NO) [7]. Activation of plant defense systems is often associated with a fitness cost. However, the extent of the fitness penalty differs largely between PRIs and is also dependent on the growth environment [8,9] (Figure 1).

Priming is a commonly used term. It can be seen as a special case of IR response after the application of PRIs, in which plants react more rapidly to a stress because they are in an induced state [10]. The general notion is that priming has a low fitness cost and that only subtle changes take place when induction occurs by a PRI. However, at least the latter can be questioned since there are now several genome-wide studies showing that substantial changes in the expression of genes, proteins and metabolites take place (for example [11,12]). Based on “-omics” studies, it was recently suggested to divide the priming phenomenon into three different stages: a “priming phase”, a “post-challenge primed state” and a “transgenerational primed state” [13]. In the first stage, which is true for all types of PRI responses the levels of various transcripts, proteins and metabolites are altered, putting the plant in a standby state. In the post-challenge primed state reactions combating the stressor are induced more rapidly. The last stage, the transgenerational primed state, has been reported recently in several plants [14,15]. Plants generated from seeds from primed parental plants have a priming memory and are thus able to react more rapidly when challenged by a pathogen.

In modern agriculture, two main strategies have so far been employed to combat crop pathogens: resistance breeding and application of chemical pesticides. In potato and tomato, traditional breeding has been used to introduce resistance genes from wild relatives for which there are available resistance sources. However, this is time-consuming and since many pathogens adapt rapidly, there are numerous examples were the resistance based on introduced resistance genes has been overcome rapidly if not combined [16]. Likewise the use of pesticides targeting cellular processes to hinder the growth of the pathogen can cause resistance, such as highly problematic metalaxyl resistance in *Phytophthora infestans* [17,18]. 

A third way would be to enhance the plant’s own innate immunity by PRIs, which has some appealing aspects. Since PRIs are working indirectly on the pathogen through the plant’s innate immunity, the PRI does not need to be directly toxic to living organisms, which is the basis of plant pesticides. Thus, PRIs have the potential to be more environmentally sustainable with less impact on human health. Most farmers in developing countries do not use appropriate safety equipment during the application of harmful chemicals [19]. Non-toxic alternatives in plant protection such as the use of PRIs activating the plants’ own defense could therefore come to play an even more important role in developing countries. Furthermore, many PRIs give a broad spectrum resistance, which in turn lessens the likelihood of the development of pathogen pesticide resistance [20]. For example, probenazole has been used against *Magnaporthe grisea*, the rice blast fungus, and *Xanthomonas*, causing bacterial leaf blight in rice, for more than 30 years and resistance in the pathogen has not been reported [21]. Likewise, potassium phosphite has been used in potato for many years in some tropical countries [19]. 

There is also the possibility to combine PRIs with biocontrol agents, i.e., living organisms controlling disease or pests by acting as a predator, parasite or pathogen of the disease-causing species. In addition, PRIs can complement current pesticide treatments and thereby reduce the amount of pesticides necessary for efficient control.

Future breeding programs may take the inducibility of pathogen resistance into account, something that has been discussed for some years [7]. Lately, increased attention has been given to the interaction between pests and pathogens in relation to the resistance level of the host plant, which needs to be taken this into consideration (see e.g., [3,22]). In summary, PRIs could form a future important part in integrated pest management (IPM), for example in order to increase the longevity of resistance genes or chemicals. The focus of this review is PRIs effective in the *Solanaceae* plant family, which belongs to the asterid clade of flowering plants comprising both annuals and perennials ranging from herbs to shrubs and trees. It contains many agronomical important annual crops such as potato, tomato and eggplants. The genus *Nicotiana* is both economically important and functions as models to explore basal biochemical and molecular functions of plants. *Solanaceae* has entered the genome era with potato being sequenced in 2011 [23], tomato in 2012 [24] and tobacco in 2014 [25]. There are several *Solanaceae* members in the 1001 genome project (www.onekp.com) and this will further increase our possibilities to understand immune responses in this plant family. 

We do not cover biocontrol in this review, and consequently we are also omitting organisms affecting the rhizosphere, which can trigger ISR. We want to stress that the focus of this review is on plant pathogens—bacteria, oomyctes, fungi and to some extent viruses—and not pests such as insects and nematodes. It should, however, be noted that several PRIs have been shown to affect both pests and non-pathogenic organisms. For example, β-aminobutyric acid (BABA) decreases root-knot nematode infections in potato [25]. Additionally, some PRIs have been reported to have an effect on abiotic stresses, e.g., BABA improved drought tolerance in potato [26]. In the first section we give examples of PRIs which are efficient in *Solanaceae* species followed by some mechanistic studies in this plant family together with “-omics” studies that recently have appeared. Finally, we discuss practical applications of *Solanaceae* plant resistance inducers and future prospects of PRIs.

## 2. Plant Resistance Inducers (PRIs) in *Solanaceae* Species

This section highlights PRIs shown to have disease suppressing effects in solanaceous species, divided into non-biological and bio-based PRIs. A summary of studies can be found in Table 1. It is frequently reported that these agents perform less well in direct toxicity assays compared to when applied directly onto the plant, which is an indication of that these agents induce the host plant innate immunity. However, this needs to be verified by studies *in planta*, where resistance should be proven by the subsequent inoculation of pathogens of both induced plants and non-induced control plants. Such experiments can be further verified by testing molecular markers of, e.g., SAR, or genome-wide studies of gene expression, which have come down in price and nowadays are viable options. According to these criteria several compounds are known to be PRIs in *Solanaceae*, including BABA, thiamine (vitamin B1) and 1,2,3 benzothiadiazole-7-carbothioc acid *S*-methyl ester (benzothiadiazole, BTH). These compounds are usually without direct toxicity to the pathogens. Other compounds, e.g., phosphite show a dual effect with direct toxicity with regard to certain pathogens, especially oomycetes, at the same time as it induces defense pathways after application.

### 2.1. Non-Biological PRIs

Chemically synthesized and pure compounds as PRIs have been tested for many years and here we discuss three different groups, which are successful in *Solanaceae* (Table 1).

#### 2.1.1. 2,6-Dichloro isonicotinic acid (INA), Benzothiadiazole (BTH), and 3-Chloro-1-methyl-1H-pyrazole-5-carboxylic acid (CMPA)

The interest in 2,6-dichloro isonicotinic acid (INA) and benzothiadiazole (BTH) were sparked when they were discovered to mimic the biological activation of SAR in large chemical screens in the 70’s [20]. BTH was later introduced as a plant protection agent with different trade names [82]. INA was early shown to induce stress response proteins in tomato [83]. BTH has been shown to be effective against *P. infestans* in tomato and several other species, but it has not been shown to protect potato against this pathogen with the concentrations used in the experiments [62,84]. This highlights the fact that there are variations in efficacy depending on the plant-pathogen interaction studied even if these compounds generally give broad resistance in many plant species. Both INA and BTH have been described as SA analogues both functionally and structurally. They are efficient in tobacco *NahG* transgenic plants lacking SA [85], but are more efficient than SA itself as they are more stable. Recently, it was also shown that resistance to *Botrytis cinereae* by soil drench of BTH, induced transcripts related to both SA and ethylene such as *PR1a* and *GluB* [86]. Likewise, the pyrazolecarboxylic acid derivative, 3-chloro-1-methyl-1H-pyrazole-5-carboxylic acid (CMPA), induce resistance independently of SA accumulation in tobacco [87]. CMPA remains little studied in other *Solanaceae* species.

#### 2.1.2. β-Aminobutyric Acid (BABA)

β-Aminobutyric acid (BABA), which is a non-proteinaceous amino acid, is one of the most studied PRIs. It has been shown to have a broad efficiency against virus, bacteria, fungi and oomycetes in several solanaceous plants. For example, BABA is efficient against the fungal pathogen *Colletotrichum coccodes* in pepper [27], bacterial canker *Clavibacter michiganensis* and bacterial wilt caused by *Ralstonia solanacearum* in tomato [35,37] as well as *P. infestans* and *Phytophthora capsici* in tomato and pepper, respectively [29,88]. It also enhances the resistance in tobacco against TMV [54].

BABA’s efficiency against *P. infestans* in potato is well documented [39,59,63]. BABA can cause small necrotic lesions when applied at a concentration of 1 mM in tobacco and at 10 mM in potato [53,65]. Instead of the priming effect proposed in other plant-pathogen systems including the model species *Arabidopsis* [64], a direct activation of defense responses with the formation of HR-like lesions with expression of ROS, phenolics and PR1 seems necessary to achieve an effect of BABA in the potato-*P. infestans* system [65].

Unlike many other inducers, BABA has been reported to have a curative effect, i.e., being effective also after pathogen infection [52,66]. This could be attributed to a lower sporulation rate of the pathogen. BABA has a direct toxic effect on some pathogens such as *Leptosphaeria maculans* [89], but not towards *P. infestans* [66]. Tobacco, tomato, and pepper plants express SAR signals and induce PR1a, chitinase and glucanase expression after BABA application [52,54]. Lee et al. [28] also reported that BABA-treated pepper was protected from *P. capsici* by increased cell wall appositions that may hinder hyphal growth and sporangia formation. BABA has been reported to be ineffective against powdery mildew [66].

#### 2.1.3. Inorganic Salts

Several inorganic salts have been shown to be effective against *Solanaceae* pathogens, but so far it has only been reported that silica, sulfate, phosphate and phosphite salts can lead to IR [47,90]. So called sulfur-IR has been shown to be effective against TMV in tobacco [51]. This could be linked to increased glutathione levels and release of gluthatione from mitochondria upon TMV inoculation. In potato, application of aluminum causing root stress was reported to lead to increased resistance against *P. infestans* associated with increased pathogenesis-related (PR) transcript expression and dependence on the SA and Nitrogen Oxide (NO) pathways [72].

There are a few reports on effectiveness of phosphate salts as protective agents, but little on whether they lead to IR. However, one study reports that monopotassium phosphate can induce resistance both locally and systemically against powdery mildew in pepper [30]. 

On the other hand, phosphite-based salts marketed as fertilizers, plant strengtheners or systemic fungicides have been identified as potential alternatives to conventional fungicides for solanaceous crops because of their effectiveness against oomycetes, such as *P. infestans* and *P. capsici* [31,79]. They are, for example, widely use in some developing countries because of their low risk to human health and environment [19]. Phosphites are alkali metal salts of phosphorous acid (H_3_PO_3_) [91]. Phosphite salts contain a cation, such as K^+^, Na^+^, or NH^4+^, and any of the following anions: phosphite (PO_3_^−3^), hydrogen phosphite (HPO_3_^−2^) or dihydrogen phosphite (H_2_PO_3_^−^). Phosphite salts can both induce resistance and have a direct toxic effect on the mycelial growth of oomycetes [79,92,93]. *P. infestans* and other oomycetes have considerably lower EC_50_ values (between 50 and 600 µg/mL) than pathogenic fungi and bacteria [94]. However, as pointed out by Lobato [94] it is risky to make extrapolations of the sensitivity to phosphite of these pathogens to entire kingdoms, since significant differences in the sensitivity to phosphite compounds were found within various classes of oomycetes, and even within species, as e.g., reported in [95]. 

Phosphite probably enters the cell via phosphate transporters and consequently interferes with phosphate signaling mechanisms because of its steric resemblance, which potentially could lead to indirect induction of resistance [96]. Using NMR in tobacco cell cultures, Danova-Alt et al. [97] showed a cytoplasmic accumulation of phosphite in a phosphate deprived state, whereas this was shifted to vacuolar compartmentalization in phosphorous pre-loaded cells. 

### 2.2. Bio-Based PRIs

Bio-based inducers are derived from living organisms. There are several examples of the successful use of these as PRIs in *Solanaceae* species (Table 1). For reviews on general bio-based PRIs see recent papers by Burketova et al. [98] and Wiesel et al. [99].

#### 2.2.1. Intrinsic Compounds Used as PRIs 

There are numerous reports on exogenous application of endogenously produced plant hormones effective as PRIs. SA induces SAR in pepper [41], and gives protection against *R. solanacearum* in tomato [48]. In vitro potato cultures supplemented with SA have been shown to be more resistant against *Dickeya solani* [70], and IAA has been found to be effective against *Fusarium oxysporum* in tomato [42]. In tobacco both *cis* and *trans*-zeatin isomers were shown to suppress infections by the bacterial pathogen *Pseudomonas syringae* [50], suggesting that also these cytokinin growth hormones can induce plant defense

Nitric acid and reactive oxygen species (ROS) are important signaling molecules during plant-biotic interactions [100]. For example, treatment with l-arginine, a precursor of NO biosynthesis, influenced IR and reduced lesion size caused by *B. cinerea* in harvested tomato fruits [43]. Furthermore, NUBS-4190, a new synthetic bis-aryl-methanone compound without direct antimicrobial properties, elicits NO production in *Nicotiana benthamiana* and was shown to be effective against *P. infestans*. Interestingly, this was associated with up-regulation of *NbPR1a* transcripts but without ROS generation and HR [58]. This compound was later also demonstrated to increase resistance against *P. infestans* in potatoes, but, as in many other cases for potato the resistance was associated with ROS generation and lesion formation [101].

Hexanoic acid is produced by some plants and forms one of the branches of the oxylipin pathway. It is known to have a direct antifungal effect, but has later been proven to also induce resistance in, e.g., tomato against *B. cinerea* and *P. syringae* [44,45,102]. Lately there have been several genome-wide studies of the IR effects of hexanoic acid (see Section 3.2).

#### 2.2.2. Bacterial Elicitors

The recent review of Burketova et al. [98] presents an extensive collection of bacteria-derived compounds eliciting defense responses in plants. From this review and other literature it becomes evident that in relation to tobacco and tomato, few effective bacterial elicitors have been identified for potato. One exception is the oligosaccharide curdlan derived from a soil bacteria, which induces resistance mechanisms and reducing *P. infestans* lesions by 50% in potato [73]. Upon curdlan application it was also shown by 2D gel electrophoresis that 10 proteins, including a serpin and an endochitinase known to be active in plant defense were up-regulated. Similarly, application of a bacterial harpin protein derived from *Erwinia amylovora* reduced *P. infestans* infection in tomato [46]. Li et al. [55] investigated IR in tobacco by another harpin protein (PopW) obtained from the bacterium *R. solanacearum*. PopW-IR against TMV depended on SA, and the authors reported control efficacies of 81%–97% at low concentrations of PopW (25 µg/mL) in both treated and neighboring leaves in greenhouse conditions and a 45% efficacy in field trials. We could not find any data regarding treatments of potato. Both yield and leaf quality was improved by the PopW treatment. In addition, bacterial volatiles organic compounds (VOCs) have been shown to be effective against bacterial spot disease caused by *Xanthomonas axonopodis* pv. *esicatoria* and up-regulate a *PR1* transcript in pepper [32]. Veluchamy et al. [103] screened the variation in heirloom tomato lines for the responsiveness to three bacterial peptide elicitors and observed large differences between genotypes. However, there was not a clear correlation between responsiveness and level of resistance to bacterial-speck disease caused by *P. syringae* pv. *tomato*. Another study showed that purified lipopolysaccharides from *Pectobacterium atrosepticum* and *Pseudomonas corrugata* could induce defense responses in cell suspensions of tobacco, tomato and potato [104]. Biotensides from bacteria have also been reported to induce some resistance with increased PR protein accumulation towards *P. infestans* in potato [105].

#### 2.2.3. Fungal Elicitors

Chitosan has direct anti-microbial activity at the same time as it triggers IR by acting as a pathogen-associated molecular pattern (PAMP; [106]). This PRI is produced by deacetylation of chitin from crustaceans. Chitosan improved resistance against *R. solanacearum*, *F. oxysporum* and *P. capsici* in tomato [47,48,107]. In pepper and tomato it induces SAR and stress-related pathways [41,47]. A comparison of the binding capacity and activity of two oceanic oligosaccharides chitosan oligosaccharide (COS) and alginate from brown algae, showed that COS clearly had a higher bioactivity in tobacco cells [108].

The protein PeaT1 from *Alternaria tenuissima*, produced in *Escherichia coli*, and thereafter used to treat tobacco led to systemic resistance and significant reduction in both number and size of TMV lesions on leaves [56]. Reduction in TMV concentration was also confirmed by qPCR. Furthermore, peroxidase activity, lignin content and the expression of *NtPR1a* and *NtPR1b* increased significantly after PeaT1 treatment.

Pen, which is an aqueous extract of the mycelium of the ascomycete *Penicillum chrysogenum*, is another example of a fungal-derived PRI. The mycelium of this fungus is obtained as a by-product from penicillin production and is thus relatively cheap and available in large quantities, both prerequisites for a potential use in practice. It has been shown to work in several crop systems, and has for example a protective effect against *P. infestans* in tomato [109].

#### 2.2.4. Plant Extracts (Botanicals)

Botanicals and plant-derived pure substances have been investigated extensively, but their use is often hampered by the high cost, limited availability of the raw material or instability of the product. It should be noted that glucans such as laminarin from brown algae commonly used as botanicals do not seem to be very effective in solanaceous species [110]. Similarly, it is at present unclear whether the PRI saccharin and its derivative probenazole can induce resistance in *Solanaceae*.

On the other hand, treatments with unsaturated fatty acids such as arachidonic, eicosapentaenoic, linoleic, linolenic and oleic acids lead to some resistance to *P. infestans* in potato [74]. Vitamin B1 (thiamine) and vitamin B2 (riboflavin) have also been shown to induce SAR in tobacco [111,112], but their role needs to be better studied in tomato and potato. 

Biochar, charcoal used for agricultural purposes, has been reported to improve crop performance by leading to enhanced nutrition uptake by the plant and recruitment of beneficial bacteria. When added to soil, biochar has been shown to be effective in pepper and tomato against grey mold (*B. cinerea*) and powdery mildew (*Leveillula taurica*) [33]. Using tomatoes deficient in JA, ET and SA metabolism, it was shown that JA plays an important role for biochar-mediated resistance indicating the involvement of ISR [40]. 

Sugar beet extract (SBE), derived by ethanol extraction from an abundant plant waste product, has been shown to induce partial resistance against *P. infestans* in potato under greenhouse conditions [71]. Treatment with SBE in three potato genotypes, differing in their level of resistance, resulted in reduction of the size of the infection lesions in a pattern similar to that seen after application of BABA. Lower sporangial production was also observed on SBE-treated leaves, whereas the reduction in sporangial production in comparison was more pronounced after BABA treatment. SBE had no toxic effect on the hyphal growth of the pathogen or on the germination of sporangia. Instead, SBE triggered induction of PR proteins, which suggested that the protection conferred by SBE is through IR. Several phenolics and salts were found in the SBE that may contribute to the defense response.

A plant-derived fructooligosaccharide oligomer isolated from burdock roots induces the expression of markers of SAR, and SA levels increased in tobacco. Tobacco plants displayed increased resistance against TMV with a 7-fold decrease in virus levels and no visual signs of infection [57]. It has similar effects in tomato fruit and gives post-harvest protection against *B. cinerea* [49].

## 3. Induced Resistance Mechanisms in *Solanaceae*

In the past, most molecular studies of IR in solanaceous species have been limited to exploring the expression of a handful of transcripts as markers in conjunction with the application of the studied PRI. While recent genome-wide studies indicate activation of a broad range of defense responses after application of PRIs, the functional roles of the *Solanaceae* genes involved in IR largely remain unknown. However, an exception is *Nicotiana benthamiana*, which has been used extensively as a model system for molecular studies of gene functions in plant defense. It provides an easy system for transient expression or silencing of genes and is highly suitable for the lab environment. In tobacco, functional assessment has mostly concerned exogenous genes from other species but also some tobacco genes related to plant defense and IR. For extensive reviews on resistance genes and gene-to-gene resistance in *Solanaceae* species, see for example recent publications by Rodewald and Trognitz [113] and Ercolano et al. [114].

Lately, “-omics” studies have emerged giving a broader picture of molecular changes after application of PRIs. These studies have pinpointed that often major changes in transcriptomes, proteomes and metabolomes take place and that many, complex processes are at play [13]. 

Another important line of work concerns comparative genomics and transcriptomics to infer knowledge from model species to the *Solanaceae* species and other inter-species comparisons. Early work was done by comparing potato defense genes from EST collections to the *Arabidopsis* genome [115], but these types of studies can now be done more systematically by comparing the full genome sequences of potato, tomato and tobacco. For example, based on sequence identity a candidate of the AtDIR1 protein, which in *Arabidopsis* functions in the signal transduction of SAR, has been suggested to have a functional homologue in tomato localized in the phloem sap [116]. Frades et al. [117] recently used RNA-seq to assembly and compare transcriptomes of three potato clones with three wild relatives of potato in order to find and characterize transcript families correlating with the level of resistance to *P. infestans*. A similar approach can be used to identify putative IR factors.

### 3.1. Genes Underlying Induced Resistance

A number of studies have reported *Solanaceae* transgenic lines with an altered ability to induce resistance. For example, a transgenic potato deficient in expression of a plastidic ATP/ADP transporter (StAATP1) leading to less starch but higher levels of glucose was more resistant to *Alternaria solani* and could raise a more efficient IR after treatment with *P. sojae* cell wall extracts leading to delayed infection by *P. infestans* [118]. In this experiment, grafting between potato deficient in *StAATP1* expression and wild-type plants indicated the presence of a signal that is generated in *StAATP1* deficient rootstocks inducing increased resistance in wild-type leaves.

There are a few examples where well-known markers for IR have been studied mechanistically in the *Solanaceace* family. In pepper, the receptor CaLRR1 was shown to interact with and be a negative regulator of the pathogenesis-related protein CaPR4b, which is required to trigger cell death [119]. Furthermore, a receptor-like protein kinase, CaPIK1, and a class IV chitinase were shown to interact, and co-silencing of these led to increased susceptibility of *Xanthomonas campestris* pv. *vesicatoria* and lowered induction of cell death response, ROS and NO bursts and expression of defense response genes upon infection [120].

Many components of plant hormone biosynthesis and signaling influence responses of PRIs such as SA and JA/ET hormone pathways. Potato allene oxide synthase (StAOS2) is well-characterized and essential for biosynthesis of JA. RNAi-mediated silencing of *StAOS2* in potato drastically reduced JA production and compromised potato late blight resistance. To test the natural variation of this gene, five potato *StAOS2* alleles were expressed in the *Arabidopsis* *aos* null mutant under the *Arabidopsis* *aos* promoter and tested for differential complementation phenotypes [121]. *StAOS2* alleles associated with increased disease resistance in potato complemented all *aos* mutant phenotypes better than *StAOS2* alleles associated with increased susceptibility, and putative sites affecting enzymatic activity were identified by structure models. Another example of a protein regulating hormone levels and affecting IR, is the tomato dioxigenase α-DOX2, catalyzing oxylipin production from fatty acids. This protein was shown to be required for maintenance of the enhanced protection conferred by the PRI hexanoic acid against *B. cinerea* [122]. α-DOX2 deficient tomatoes accumulated less callose and had lower levels of JA and 12-oxo-phytodienoic acid (OPDA) upon infection. Furthermore, SA-deficient *NahG* tomato plants showed that low SA levels increased resistance to *B. cinerea*, but plants were at the same time unable to display resistance after hexanoic acid treatment, indicating that SA is necessary for this PRI to function.

The role of SA in potato and its involvement in SAR signaling have been an ongoing debate because, unlike tobacco and *Arabidopsis*, potato is considered to display high levels of endogenous SA. Manosalva et al. [123] could, however, show that repression of *StMes1*, the potato ortholog of tobacco SA-binding protein 2 (*NtSABP2*), hampered SAR after *P. infestans* infection. StMes1, like NtSABP2, indeed seems to be required to convert the SAR signaling molecule methyl salicylic acid (MeSA) into biologically active SA in distal tissues. *StMes1* repression was associated with elevated levels of MeSA and reduced expression of PR genes in untreated distal tissue, which are signs that there are similarities in SAR signaling between potato and tobacco.

Suppressing the expression of the MAPK phosphatase, *NtMKP1*, in tobacco enhanced the resistance against both the necrotrophic pathogen, *B. cinerea*, and two lepidopteran herbivores, *Mamestra brassicae* and *Spodoptera litura*, showing that NtMKP1 is a negative regulator of resistance to necrotrophic pathogens as well as Lepidopteran herbivores [124].

Lipid signaling also plays a vital role in plant defense. For example, virus-induced gene silencing (VIGS) suppression of two lipid transfer protein genes, *CaLTPI* and *CaLTPII*, led to enhanced susceptibility to *X. campestris* pv. *vescatoria* and pepper mosaic mottle virus in pepper [125]. On the other hand, the constitutive expression of these two genes in tobacco plants enhanced the resistance to oomycete pathogen, *Phytophthora nicotianae* and bacterial pathogen, *P. syringae*, indicating that the effect in a certain signaling pathway can have contrasting effects depending on the type of pathogen.

There are few examples of molecular studies across *Solanaceae* species. The microrchidia proteins, MORCs, which contain a GHKL ATPase domain, are involved in transcriptional gene silencing in *Arabidopsis*. Manosalva et al. [126] silenced the expression of the corresponding *MORC1* homologs in potato, tomato and *N. benthamiana*. Basal resistance to *P. infestans* was compromised in *StMORC1*-silenced potato, whereas silencing in tomato and *N. benthamiana* instead enhanced basal resistance to the same pathogen. Interestingly, by domain-swapping and mutational analyses the authors showed that the C-terminal region caused the species-specific effects.

The molecular effects in Solanceace of some pathogen-derived elicitors known to induce resistance have been tested. The oomycete elicitor cryptogein produced by *Phytophthora cryptogea* is well studied in tobacco, which is a non-host for this pathogen. For example, the efficiency of cryptogein-IR is light-dependent, and the transcriptional response was shown to be altered depending on the light conditions [127]. Furthermore, the phenylpropanoid metabolism is altered in tobacco cell suspensions after treatment [128]. The sterol-binding ability of cryptogein has been connected to its efficiency to induce resistance in tobacco. However, in a study using site-directed mutagenesis producing cryptogein variants with different binding abilities, the authors could not clearly correlate efficiency of IR with domain function [129]. Elicitin-like proteins oligandrins can induce resistance in many plants. For example, transiently expressed in tomato leaves, *Oli-D1* and *Oli-D2* induced resistance against *B. cinerea* and activated the expression of a set of genes involved in the JA/ET-mediated signaling pathway [130]. By VIGS the same authors were also able to show that the signaling regulatory genes *NbSgt1* and *NbNpr1* were required for *Oli-D1* and *Oli-D2* to induce HR in *N. benthamiana*.

### 3.2. “-Omics” Studies on the Effect of PRIs in Solanaceae 

During the last years more than a handful studies have investigated the global effects of PRIs in *Solanaceae* by employing microarrays and RNA-seq for transcriptome analysis and mass spectrometry for protein and metabolite analyses.

To elucidate molecular defense responses activated by BABA in potato, a genome-wide microarray was used in combination with label-free quantitative proteomic analysis of the apoplast secretome [11]. Apart from this study, one more limited transcriptome analysis based on cDNA-AFLP investigated the effects of BABA-IR in potato. This study identified about 60 differentially expressed transcripts with a clear overlap of genes affected by BABA and *P. infestans* inoculation [131]. The genome-wide study by Bengtsson et al. [11] identified over 1000 transcripts with changed expression after BABA treatment and increased abundance of a large number of secreted proteins. Several processes related to plant hormones and amino acid metabolism were affected and several defense proteins were up-regulated. However, unlike in *Arabidopsis*, no clear change in expression of abscisic acid (ABA) responsive genes was detected in potato upon BABA application. In potato, BABA has previously been shown to involve SA-dependent IR against *P. infestans* [67]. A strong down-regulation of the biosynthesis of sterols and steroid glucoalkaloids, in contrast to the up-regulation of important enzymes involved in sesquiterpene phytoalexin biosynthesis, was also evident.

In potato leaves, a comparative analysis of total protein extracts using two-dimensional electrophoresis and mass spectrometry revealed a core set of 25 proteins all accumulating after BABA, γ-amino butyric acid (GABA), INA or Laminarin treatments. Identification of 18 of the 25 proteins showed that they mostly were involved in primary metabolism [132]. Whereas several components of BABA signaling have been identified in *Arabidopsis*, including the dependence on a cyclin-dependent kinase-like protein [133] and the possible function of an aspartyl-tRNA synthetase as a receptor [134], none has been identified in *Solanaceae* species and *Solanaceae* homologs remain to be explored.

The importance of the SA pathway in phosphite-IR has been demonstrated in *Arabidopsis* [135]. In potato it has been reported that phosphite leads to excessive accumulation of hydrogen peroxide and PR1 transcripts [80], which is associated with SA signaling. A time-course study of phosphite applications in potato by genome-wide microarrays did not detect up-regulation of *PR1* transcripts, even if phosphite caused a major, but transient, change of the transcriptome within 24 h [75]. Instead, genes associated with defense, wounding and oxidative stress were found to constitute the core of the phosphite response, together with changes in primary metabolism and cell wall-related processes. Interestingly, there was an overlap of 40% between phosphite- and BABA-regulated transcripts, indicating that similar processes are affected upon induction of resistance even though the effect of BABA on the transcriptome lasts longer and goes on for at least 48 h [11,75]. In a study of soluble proteins from potato leaves treated with the phosphite product Confine™, Lim et al. [77] found increased abundance of proteins involved in SA-dependent defense responses, reactive oxygen species (ROS) and calcium-dependent pathways, whereas proteins involved in amino acid and starch metabolism were down-regulated. They suggested that phosphite triggers a HR response that is responsible for defense against *P. infestans* in potato. Taken together, it seems like that multiple defense pathways are rapidly induced by phosphite treatment for enhanced resistance in local tissues.

Ghareeb et al. [136] showed by genome-wide expression analysis by microarrays in tomato that silicon affects gene expression also at the priming stage giving protection against *R. solanacearum*, which causes bacterial wilt. This was followed up by Kiirika et al. [47] using both silicon and chitosan treatments to induce resistance against *R. solanacearum*. Gene expression analysis conducted at 72 h post inoculation via microarrays revealed regulation of 204 and 126 genes in two genotypes, respectively, with the majority classified into the categories defense-related, signal transduction and transcription.

Genome-wide studies of elicitor treatments can be an efficient way to screen for new candidates important in plant basal defense. In tomato, RNA-seq was used to identify genes that were induced by elicitors but repressed by effectors by comparing gene expression patterns after application of bacterial flagellin or inoculation of *P. syringae* [137]. Of the 92 protein kinase-encoding genes identified in this experiment, 33 were subjected to VIGS in *N. benthamiana* and tested for involvement in pattern-triggered immunity (PTI). This led to the identification of a cell wall-associated kinase (WAK) gene (*Solyc09g014720*) to be necessary for PTI-associated cell death suppression.

The role of the putative elicitor fructo-oligosaccharide in tobacco was investigated with RNA-seq and this analysis showed that several hundred genes were differentially regulated upon treatment. These genes were associated with SA, JA/ET, ABA and gibberellin acid (GA) regulation as well as to production of secondary defense-related metabolites [138]. This general change in the transcriptome is similar to that seen after treatment with BABA and phosphite in potato, with the exception of ABA-related transcripts.

Hexanoic acid IR has been studied on both the transcriptomic and metabolic levels in tomato in relation to its protective effect against *B.* *cinerea* [12,139]. A marked metabolic reprogramming was associated with resistance triggered by hexanoic acid where several citrate and other primary metabolites were down-regulated.

## 4. Transgenerational Effects of Induced Resistance

In recent years a growing number of studies have identified transgenerational effects of IR to pathogens (reviewed in [14,15]). Transgenerational effects can be caused by epigenetic changes, e.g., DNA methylation [13,140,141,142], which often lasts 2–5 generations for 50% of the methylatable sites [143,144,145]. Recent studies also suggest that increased expression of defense genes in the next generation can be caused by posttranslational histone modifications [142]. Moreover, various primary and secondary metabolites, enzymes and hormones are associated with the transgenerational stage of IR [13]. The majority of studies that have investigated transgenerational effects of IR have been conducted with *Arabidopsis* challenged by pathogens or treated with PRIs such as BABA (e.g., [146]). Transgenerational effects have also been detected in solanaceous species, including TMV-infected *Nicotiana tabacum* [147,148] and BABA-treated *Solanum physalifolium* [149]. In *N. tabacum* resulted TMV infection in resistance to the virus as well as to bacterial (*P. syringae*) and oomycete (*P. nicotianae*) pathogens in the next generation. A broad effect on a range on pathogens has also been seen in *Arabidopsis* [146]. In *N. tabacum* the transgenerational effects were connected with changes in metabolites, including increased levels of amino acids and sugars [150]. 

Even though epigenetic variation have been found in studies of wild populations (*Viola cazorlensis*, [151]), few have investigated transgenerational effects of IR using plant material collected directly from the wild. Such studies can be important to learn about the adaptive significance of transgenerational IR, e.g., a capacity of rapid change in response to altered environmental conditions (see [152,153]). Studies involving wild relatives of important crop species can also improve our understanding of factors influencing abundance of economically important pathogens in the agricultural landscape as well as be of significance for developing breeding material. A recent investigation of *S. physalifolium*, a wild weed that is related to potato and highly susceptible to *P. infestans*, showed that BABA-induction resulted in transgenerational IR to *P. infestans* [149]. Unlike what has been described in *Arabidopsis* [146], an additional BABA treatment in the first offspring generation did not increase the transgenerational effect in the second offspring generation. Interestingly, BABA treatment can also lead to higher levels of defense to *P. infestans* in the next vegetative generation in potato [68]. It is clear that it would be valuable with more studies on transgenerational effects in both cultivated and wild *Solanaceae* species, also including identification of involved mechanisms. For example, it could be of interest to study how combined stresses, e.g., pathogen infection combined with abiotic stress or herbivory, would influence transgenerational IR. A recent study on the wild *Solanum dulcamara* showed that both water stress and herbivory increased abscisic acid (ABA) and JA levels, making drought-stressed plants more resistant to attack by *Spodoptera exigua* larvae [154].

## 5. Induced Resistance in Field and Practice

Using non-toxic PRIs in agriculture to induce the host plant defense is an attractive concept, since it can contribute to reduce exposure of conventional pesticides to the environment, farmers and consumers. PRIs might also prove to be easier than pesticides to combine with organisms used as biocontrol agents. Additionally, they might extend the time that resistance genes are effective. Some of the PRI compounds are relatively cheap. Still, with a few exceptions PRIs remain little used in practice in spite of numerous laboratory studies and field trials. In solanaceous species, PRIs are used commercially in greenhouses and to some extent in the field. For example, phosphite has been used against late blight in potato mainly in developing countries [19]. In a landmark study, Cruickshank and Mandryk [155] applied a weak pathogen strain of *Peronospora hyoscyami* in field-grown tobacco plants by stem injections to trigger SAR against subsequent inoculations of the same pathogen.

A number of considerations need to be taken into account when employing IR in the field [156]. One of the largest concerns is the often relatively low efficacy of protection ranging from between 20%–85% using PRIs in a field setting [2]. The variation in efficacy depends on a number of factors such as environmental conditions and presence of stress factors. In order to activate IR in the field using PRIs, the initial level of plant defense activity needs to be low. However, in a multi-stress environment, it is still unclear to what extent plant defense is already activated. However, our data suggest that only around 30% of the field samples from wild and cultivated *Solanum* spp. in Sweden expressed PR proteins, suggesting that plant defense is not constitutively on [157]. This holds promise for PRI strategies, although towards the end of the season at least in potato without disease symptoms, we detected more frequent activation of immuno-responses that might indicate that relatively early treatments could be more efficient.

Crosstalk in plant signaling pathways is also an issue of concern where PRIs might be effective against one type of pathogen, but actually increase susceptibility to another. This is a key issue in a field setting, which represents a multi-stress environment with many classes of pathogens and pests, as exemplified by the established distinction between biotrophs inducing SA-related pathways and necrotrophs and insect herbivores inducing mainly JA/ET-related pathways. In general, there is a need to move molecular studies from laboratory to field settings and also try to incorporate as much knowledge as possible from model species into crops [158]. For example, in a field experiment by Thaler et al. [36] tomato plants treated with BTH exhibited reduced symptoms of bacterial speck caused by *P. syringae* compared to non-treated plants. However, plants were at the same time more susceptible to herbivore damage caused by larvae of the beet armyworm (*Spodoptera exigua*). Interestingly, several PRIs such as BABA and phosphite activate many different pathways [11,75], which may explain protection against several different types of pathogens.

In summary, IR functions towards a broad spectrum of pathogens and many potential PRIs are available. Before using these inducers in control strategies their functionality under field or greenhouse condition must be assessed, since these environments provide a variety of combined biological and physical factors hard to test in the laboratory. In addition, cultivar dependability, optimal timing, method of application for each target crop, dose and metabolic costs must be considered (reviewed in [2]). There is still a great lack of these kinds of studies.

## 6. PRIs Tested in Solanaceous Species in Field Conditions

BTH was introduced in 1996 as a PRI under several trade names [82]. A number of studies have shown the importance of BTH-IR in field conditions. For example, BTH applied at a rate of 50 g/ha provided field protection against fungi, virus and bacteria in tobacco plants which lasted long without negative effects on the plant growth and yield [84]. BTH-IR was also effective against bacterial spot, early blight, and bacterial speck in tomato, bacterial spot in pepper and wild fire, blue mold, frogeye leaf spot, brown spot, tomato spot wild virus and Rhizoctonia leaf spot in tobacco, and lead to increased PR1 expression [138].

Yi et al. [159] observed an additive IR effect against bacterial spot in pepper, caused by *X. axonopodis* pv*.* *vesicatoria*, using a combination of BTH and an endophytic plant growth-promoting rhizobacteria, *Bacillus pumilus* INR7. This is an example of improved efficacy of combined treatments using a chemical PRI (triggering SAR) and a biological agent (triggering ISR). However, similar experiments with tobacco revealed no additive effect under field conditions.

Huang et al. [160] reported that field efficacy of BTH in tomato depended on its application frequency and rate. Weekly applications of BTH at concentrations of 75–200 µM gave better protection against bacterial spot (*Xanthomonas* spp.), compared to treatments performed every second week. Both weekly and bi-weekly applications of BTH reduced disease severity compared to standard copper treatment.

Induction of resistance by BABA is dependent on how it is applied to the target plant, the developmental stage and cultivar used. For example, in tobacco stem injection of BABA was ineffective and failed to accumulate PR proteins, whereas foliar application of BABA 30 days after crop emergence showed 60% efficacy against late blight in moderately susceptible potato cultivars and 20% in highly susceptible ones [54,60,61].

Cohen [66] reported a 60% control of late blight after BABA treatment of field-grown potato. However, later Liljeroth et al. [63] found less than 20% reduction in late blight disease in field trials after weekly applications of BABA, which is not enough for effective protection on its own. Instead, considering combinations with conventional pesticides could be a way to incorporate PRIs into plant protection strategies. A synergistic interaction of BABA with fungicide metalaxyl was reported to control blue mold in tobacco, and with mancozeb in controlling *P. infestans* in potato [69] in greenhouse trials. Liljeroth et al. [63] tested combinations between BABA and the fungicide Shirlan (a.i. fluazinam) in field experiments and found an additive but not synergistic effect between the two agents. A low dose of BABA could be mixed with a reduced dose (80%) of Shirlan, still obtaining the same level of protection as full dose of fungicide. However, half the dose of fungicide mixed with BABA resulted in somewhat higher infection rates compared to full dose of fungicide [63].

An important question regarding the use of PRIs is the durability of the effect, for which there to our knowledge are very few studies performed under field conditions. According to Cohen [39] a single dose of BABA on tomato was reported to be effective for 12 days against *P. infestans*, and the durability seems to largely depend on the pathosystem [66]. In greenhouse trials, Liljeroth et al. [63] found a durability of BABA resistance in potato of 4–5 days.

Phosphites can be applied in different ways and the application method depends on the crop-pathogen combination, but foliar application is most common [47]. However, other techniques like fertigation, i.e., trunk spray, trunk injection, trunk paint, in-furrow as well as root or soil drenches can also be used [90]. A number of field studies have shown the importance of phosphite-IR against various disease-causing pathogens in solanaceous plants. For example, foliar application of fosetyl-aluminium at 3 kg a.i./ha reduced late blight infection and tuber blight in potato [61]. Field trials over several years have shown that foliar applications of phosphite reduced the susceptibility of tubers to *P. infestans* [76,161]. Post-harvest application of phosphite was also found to be effective in controlling tuber blight caused by *P. infestans* in stored potato tubers [79]. Combinations of phosphite and conventional pesticides like mancozeb in different ratios can provide effective control. Combined application of 1.3 kg/ha of Mancozeb with 2 kg/ha of phosphite gave better protection against potato tuber blight than the conventional pesticide [161]. An integration of spray programs involving phosphite and reduced doses of systemic or non-systemic fungicides was suggested to suppress foliar and tuber blight infection as well as overwintering survival of *P. infestans* in potato tubers.

Generally, there are very few published field trials comparing the effect of phosphite with conventional fungicides. Deliopoulos et al. [90] reviewed studies where the effects of various salts were compared with fungicides alone or in combination. Kromann et al. [19] made a meta-analysis of several field trials in tropical countries and found that phosphite applied in rates of around 2.5 g a.i./L gave similar levels of protection as two conventional contact fungicides, mancozeb and chlorothalonil. The level of control appeared to be relatively independent of the geographical location of the field. In field trials by Mayton et al. [162] it was found that phosphite controlled late blight to a similar degree as chlorothalonil and even better against tuber blight. Wang-Pruski et al. [163] also reported that phosphite provided protection against potato late blight under field conditions, but to a less extent than chlorothalonil. Interestingly, the most efficient control was obtained with a combination of phosphite and chlorothalonil.

**Figure 2:**
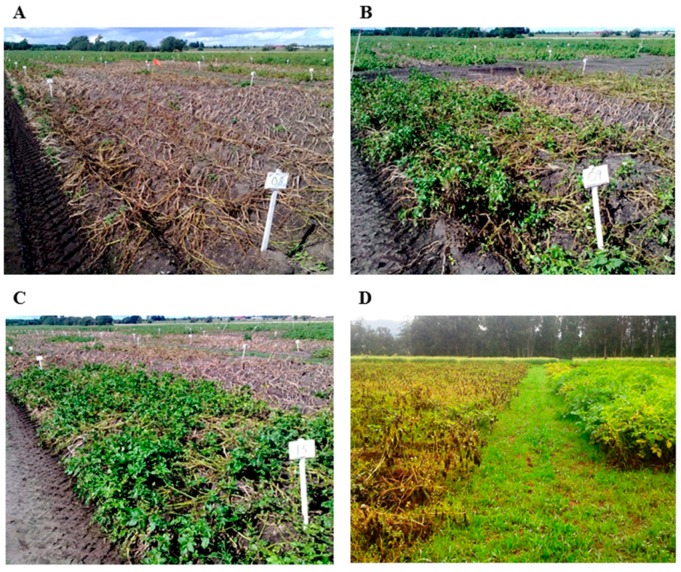
Phosphite is efficient towards late blight in potato under different growing conditions. Potato late blight infection in Sweden (**A**–**C**) and in Ethiopia (**D**). Untreated (**A**), fungicide Shirlan at recommended dose (**B**), a combination of potassium phosphite and half dose of Shirlan (**C**) and untreated to the left and combination of Rodimil and potassium phosphite to the right in the same photo (**D**). The combination with reduced dose of fungicide had the best effect against late blight.

Liljeroth et al. [76] studied the effect of foliar phosphite applications, alone and in combination with modern fungicides on both late blight and tuber blight. Potassium phosphite in combination with half the dose conventional fungicides gave the same level of protection as treatments with recommended full dose of fungicides in field trials performed over 4 years. In starch potato cultivars with relative high level of resistance, potassium phosphite alone gave sufficient protection and when combined with fungicides the intervals between treatments could be significantly extended without increased late blight infections. This finding may be connected to the stability of phosphite inside plants. A study in progress in Ethiopia is testing the possibility to combine phosphite with the fungicide Ridomil gold (Figure 2). So far, results have shown that a 75% reduction of the recommended dose fungicide combined with phosphite gives as good protection against *P. infestans* as full-dose fungicide [164].

Taylor et al. [165] and Miller et al. [81] reported that foliar field application of phosphite in combination with post-harvest application gave good protection against pink rot, caused by *Phytophthora erythroseptica* during storage. Post-harvest application of phosphite can have a good effect against the spread of potato tuber blight during storage [78,166].

Plants take up phosphite and translocate it to different organs, including the tubers ([167], but are not able to metabolize phosphite and use it as a phosphorous source. That means that residues of phosphite will be present in harvested products, which may be a concern despite very low toxicity in rats (LD_50_ > 5 g·kg^−1^, Eurpean food safety authority (EFSA) 2012). Liljeroth et al. [76] found residue levels ranging from 27–205 mg PO_3_·kg^−1^ potato tuber after repeated foliar treatments with phosphite. The level depended on the rate of application and perhaps also cultivar. As far as we know there are no maximum residue levels (MRL) for phosphite in USA or Canada where phosphite based products are registered for use in e.g., potato. In Europe, phosphite is not yet registered as a plant protection agent in solanaceous crops, but used in grapes and some horticultural crops as a plant strengthener. EU presently considers the MRLs set for fosetyl-Al as applicable. Current MRLs for fosetyl-Al in potato and tomato are 30 and 100 mg·kg^−1^ (EFSA, 2014). Further analyses of residue levels are needed to determine the amounts that can be applied without exceeding MRLs. As far as we know residue levels of other PRIs have not been considered and reported in the literature.

IR triggered by Pen has been shown to be effective under field conditions against a number of plant diseases. A greenhouse experiment has also confirmed its potential in controlling late blight in tomato [168]. Furthermore, if compared with other well-known PRIs such as BABA and BTH, efficacy of Pen was equal or superior. However, as for many other PRIs there are undesirable phytotoxic side-effects.

Incorporation of PRIs into plant protection strategies would add another mode of action, which might reduce the selection pressure for development of pesticide resistance and also reduce the amount of pesticides necessary for effective control. However, in order to implement a wide use in agriculture more field trials are needed where combinations, doses and intervals are tested more carefully and adjusted to the cultivars’ resistance.

## 7. Conclusions and Future Perspectives

During the last three decades plant resistance inducers (PRIs) have received much attention as potential main players for plant protection strategies in crop management (Figure 1). However, with the exception of potassium phosphite, PRIs are currently used to a very limited extent in the major solanaceous crops potato and tomato when grown in conventional agriculture. From the literature reviewed in this article, it is clear that in many cases hypersensitive-like reactions have to form in order for the PRI to be effectual in the *Solanaceae* family.

At the end of the 1990s and after the genomic revolution in the early 2000s the mechanisms of IR have been studied in more detail at a molecular level. Still, IR mechanisms remain fairly unexplored for field crops including solanaceous crops. Untargeted “-omics” studies looking at genome-wide changes are just starting to emerge along with the availability of the genomes of potato, tomato and tobacco. Such studies can form a good basis to better understand PRI response mechanisms in *Solanaceae.* From these initial studies it has become obvious that PRIs result in complex responses including many cross-linked and sometimes even antagonistic pathways. So far, most attention has been given to factors up-regulated as possible efficient underlying resistance mechanisms. However, current studies now also considers susceptibility factors. These factors should apply to responses to PRIs as down-regulation of susceptibility factors might explain the increased resistance observed. “-omics” studies could also be used to understand cases where PRIs have been shown to have negative effect on protection against certain *Solanaceae* pathogens. “-Omics” data could also be used to predict if multiple effects are to be expected in a multi-stress environment. Potential negative effects of complex blends vs. simple compounds could be an additional important issue for the future. Global untargeted metabolomics is still a challenge in any system and the metabolic shifts after challenge with PRIs observed in model species need yet to be better studied at a larger scale in *Solanaceae*. Changes in the composition of both primary and secondary metabolites can be a key to why resistance is achieved.

It is now clear that there are not only strong between-species differences in PRI responses but also that effects are strongly dependent on genotype within species. Further studies on the underlying molecular differences in PRI responses are much needed. Additionally, identification of markers for PRI responses to assist targeted breeding would be valuable. Genotype-specific differences at the molecular level have not yet been studied systematically in solanaceous crops. A global gene expression study of seven *Arabidopsis* accession found significant levels of natural variation in response to exogenous SA [169], suggesting that similar results might be expected also in crop species. Several transcript biomarkers associated with IR have been proposed. In a commendable effort by Bernonville et al. [170] used 28 transcript markers to screen for novel PRIs and found correlation between the level of defense activation and protection efficiency in controlled conditions. These types of biomarker approaches have future potential and could also be used to identify genotypes with strong capacity for IR in *Solanaceae*.

Lately, the phenomenon of transgenerational as well as transvegetative PRI effects have been described in several species including tobacco and potato. This holds promise for its use in seed and seed potato production to give field crops a stronger basal resistance potentially already from the seed and tuber stage. 

To implement the use of PRIs in field grown crops they have to be integrated into plant protection strategies, involving also host plant resistance and probably chemical plant protection methods. More studies on how PRIs best can be applied in the field (e.g., season, frequency and dose) for maximum efficacy against disease are necessary. By combining methods some of the problems with PRIs, e.g., the variation in efficacy, may be levelled out and possible to handle. For an efficient integration of PRIs in integrated pest management (IPM) it will be important to better understand and take host-specific IR responses into account. For IPM strategies, it is also necessary to test the effects of the PRIs on the agri-system as a whole, for example, ensuring that beneficial parasitoids, or other biocontrol agents are not negatively affected. In general, side effects of the use of PRIs remain little studied. One important aspect is the PRIs’ effect on nutrient content and composition of crops since many defense pathways and IR responses are closely related to the production of nutrients important to humans. For example, it has been found that l-ascorbic acid (vitamin C) content in tomato varied depending on oxidative status of the fruit [171], indicating that future PRI studies in crops should include also this aspect.

## Figures and Tables

**Table 1:** A summary of studies of plant resistance inducers (PRIs) in *Solanaceae* (see text for details).

*Solanaceae* Species	Plant	Inducer	Protection against	Organism	Reference
*Capsicum annuum*	Pepper	BABA	*Colletotrichum coccodes*	Fungus	[27]
*Capsicum annuum*	Pepper	BABA	*Phytophthora capsici*	Oomycete	[28,29]
*Capsicum annuum*	Pepper	Monopotassium phosphate	*Powdery mildew *	Fungus	[30]
*Capsicum annuum*	Pepper	Phosphite	*Phytophthora capsici*	Oomycete	[31]
*Capsicum annuum*	Pepper	Bacterial VOCs	*Xanthomonas axonopodis*	Bacteria	[32]
*Capsicum annuum*	Pepper	Biochar	*Leveillula taurica*	Fungus	[33]
*Solanum lycopersicum*	Tomato	BABA	*Clavibacter michiganensis*	Bacteria	[34,35]
*Solanum lycopersicum*	Tomato	BTH	*Pseudomonas syringae*	Bacteria	[36]
*Solanum lycopersicum*	Tomato	BABA	*Ralstonia solanacearum*	Bacteria	[37]
*Solanum lycopersicum*	Tomato	BABA, seed treatment	*Oidium neolycopersici*	Fungus	[38]
*Solanum lycopersicum*	Tomato	BABA	*Phytophthora infestans*	Oomycete	[39]
*Solanum lycopersicum*	Tomato	BTH	*Botrytis cinerea*	Fungus	[40]
*Solanum lycopersicum*	Tomato	SA	*Ralstonia solanacearum*	Bacteria	[41]
*Solanum lycopersicum*	Tomato	IAA	*Fusarium oxysporum *	Fungus	[42]
*Solanum lycopersicum*	Tomato	L-arginine, post-harvest	*Botrytis cinerea *	Fungus	[43]
*Solanum lycopersicum*	Tomato	Hexanoic acid	*Pseudomonas syringae*	Bacteria	[44]
*Solanum lycopersicum*	Tomato	Hexanoic acid	*Botrytis cinerea*	Fungus	[45]
*Solanum lycopersicum*	Tomato	bacterial Harpin protein	*Phytophthora infestans*	Oomycete	[46]
*Solanum lycopersicum*	Tomato	Chitosan	*Ralstonia solanacearum*	Bacteria	[47,48]
*Solanum lycopersicum*	Tomato	Biochar	*Botrytis cinerea*	Fungus	[33]
*Solanum lycopersicum*	Tomato	fructooligosaccharide	*Botrytis cinerea*	Fungus	[49]
*Nicotiana tabacum*	Tobacco	Zeatin	*Pseudomonas syringae *	Bacteria	[50]
*Nicotiana tabacum*	Tobacco	Sulfur	*Tobacco mosaic virus*	Virus	[51]
*Nicotiana tabacum*	Tobacco	BABA	*Peronospora tabacina*	Oomycete	[52]
*Nicotiana tabacum*	Tobacco	BABA	*Tobacco mosaic virus*	Virus	[53,54]
*Nicotiana tabacum*	Tobacco	Bacterial harpin protein	*Tobacco mosaic virus*	Virus	[55]
*Nicotiana tabacum*	Tobacco	PeaT1	*Tobacco mosaic virus*	Virus	[56]
*Nicotiana tabacum*	Tobacco	fructooligosaccharide	*Tobacco mosaic virus*	Virus	[57]
*Nicotiana benthamiana*	Tobacco relative	NUBS-4190	*Phytophthora infestans*	Oomycete	[58]
*Solanum tuberosum *	Potato	BABA	*Fusarium solani*	Fungus	[59]
*Solanum tuberosum *	Potato	BABA	*Phytophthora infestans*	Oomycete	[39,59,60,61,62,63,64,65,66,67,68,69]
*Solanum tuberosum *	Potato	SA	*Dickeya solani *	Bacteria	[70]
*Solanum tuberosum *	Potato	SBE	*Phytophthora infestans*	Oomycete	[71]
*Solanum tuberosum *	Potato	Aluminium	*Phytophthora infestans*	Oomycete	[72]
*Solanum tuberosum*	Potato	Curdlan	*Phytophthora infestans*	Oomycete	[73]
*Solanum tuberosum*	Potato	Linoleic acid	*Phytophthora infestans*	Oomycete	[74]
*Solanum tuberosum*	Potato	Oleic acid	*Phytophthora infestans*	Oomycete	[74]
*Solanum tuberosum*	Potato	Phosphite	*Phytophthora infestans*	Oomycete	[75,76,77,78,79,80,81]

BABA, β-aminobutyric acid; VOCs, volatiles organic compounds; BTH, benzothiadiazole; SA, salicylic acid; SBE, sugar beet extract; NUBS, a bis-aryl-methanone; IAA, indole acetic acid.
